# Deciphering the origin of total estrogenic activity of complex mixtures

**DOI:** 10.3389/fnut.2023.1155800

**Published:** 2023-03-23

**Authors:** Emma Debon, Bastien Gentili, Hélia Latado, Patrick Serrant, Flavia Badoud, Marion Ernest, Nicolas Christinat, Thomas Bessaire, Benoit Schilter, Maricel Marin-Kuan

**Affiliations:** ^1^Nestlé Research, Société des Produits Nestlé SA, Lausanne, Switzerland; ^2^Bouc Bel Air, France; ^3^Lausanne, Switzerland

**Keywords:** estrogenic activity, High Performance Thin-Layer Chromatography (HPTLC), isoflavones, chemical identification, mixture assessment

## Abstract

**Introduction:**

Identifying compounds with endocrine properties in food is getting increasingly important. Current chemical analysis methodology is mainly focused on the identification of known substances without bringing insight for biological activity. Recently, the application of bioassays has been promoted for their potential to detect unknown bioactive substances and to provide information on possible interactions between molecules. From the toxicological perspective, measuring endocrine activity cannot inform on endocrine disruption and/or health risks without sufficient knowledge on the nature of the responsible factors.

**Methods:**

The present study addresses a promising approach using High Performance Thin-Layer Chromatography (HPTLC) coupled to bioassays were analyzed using the Liquid Chromatography Mass-Spectrometry (LC-MS). The estrogen receptor activation was assessed using the transcription activation Estrogen Receptor Alpha Chemical Activated LUciferase gene eXpression assay (ERα- CALUX) and the HPTLC coupled to the Estrogen Screen Yeast assay (p-YES).

**Results:**

Seven isoflavones were identified in the soy isolates. Estrogen receptor activation was assessed for both, the identified isoflavones and the soy isolates with ERα-CALUX test. Correlation between the soy isolates extracts and the identified isoflavones was shown. Moreover, p-YES revealed the presence of an estrogenic bioactive zone. Analysis of the bioactive zone through LCHRMS highlighted signals corresponding to several isoflavones already detected in the isolates as well as two additional ones. For all detected isoflavones, an estrogenic activity dose-response was established in both bioassays.

**Conclusion:**

Finally, genistein, daidzein, and naringenin were found as the most active substances. A concordance analysis integrating the analytical and bioassay data indicated that genistein and daidzein were the drivers of the estrogenic activity of these soy protein isolates. Altogether, these data suggest that the integration of HPTLC-bioassay together with chemical analysis is a powerful approach to characterize the endocrine activity of complex mixtures.

## Introduction

Over the years, the presence of endocrine disruptors in foods has raised increasing scientific, public, regulatory and industrial attention because of their potentials to adversely affect human health (1, 2). Consequently, their identification and detection are becoming a priority to estimate exposure and then conduct risk assessments. However, identifying endocrine disruptors in food is a challenging task. Foods are complex mixtures comprising thousands of chemicals, with many of them being structurally uncharacterized and among which, a number could present endocrine properties. Endocrine active chemicals found in foods can be naturally occurring substances produced by food commodities (e.g., phytoestrogens in soy), or be contaminants from various origins, including for example, agricultural practices (e.g., zearalenone, pesticides) or food contact materials (e.g., bisphenol A, non-ylphenol) (1, 2).

To detect specific substances in complex mixtures, several analytical chemistry tools and approaches have been developed, in particular the liquid chromatography coupled to high resolution mass spectrometry (LC-HRMS). The choice of the approach is driven by the prior knowledge available regarding the chemical composition of the mixture under investigation. Target analysis to detect thousands of analytically well characterized molecules, suspect screening using lists of structurally known chemicals, and/or non-target screening providing only a partial picture of chemicals present in a sample, are being applied to analyze complex mixtures such as foods (3). But the development of analytical methods adapted to the safety evaluation of complex food matrices remains a scientific and technical challenge. The identification of each peak seen on a chromatogram is resource intensive and complicated. In addition, because analytical chemistry does not provide much insight on the biological properties of the detected molecules, establishing a correlation between endocrine activity and the identified substances in a mixture appears often very difficult. Thus, developing an efficient method linking biological properties and chemical composition is key to fully understand the endocrine properties and possible associated health risks of complex mixtures such as foods.

In an attempt to improve the investigation of endocrine properties of complex mixtures, efforts have recently focused on the application of Effect-Directed-Analysis (EDA) (4–6). This approach consists of applying bioassays as analytical detectors (4). This is thought to bring significant advantages. For examples, it should allow for the detection of unexpected molecules of unknown structures and to get information on potential additive or other interaction effects. EDA has been proposed and applied to test whole food products (5, 6). However, several drawbacks have also been highlighted. Bioassays are tools which have been originally developed to study the biological properties of single chemicals in a hazard identification framework. To transfer such methods for food analysis requires following a full validation process including a thorough qualification of sample preparation (5, 6). Another limitation resulting from the use of bioassays as analytical detectors is the difficulty to interpret the results in terms of safety. Indeed, bioassay data provide information on activity. Nonetheless, endocrine activity cannot be directly translated into endocrine disruption and health risks (7). There is currently no method available to directly assess the safety of an endocrine activity. The elucidation of the chemical(s) responsible for the activity is a prerequisite for risk assessment.

In this context, High Performance Thin-Layer Chromatography (HPTLC) coupled to yeast-based assay (p-YES) (8–10) has emerged as a promising approach to tackle the difficult question of the concordance between chemical composition and endocrine activity. In this technique, mixtures are first separated by HPTLC, and then active substances are revealed with a specific bioassay conducted directly on the plate. Its feasibility has been well documented for several activities such as estrogen receptor activation (11) as well as for androgen receptor activation and inhibition (12, 13). Furthermore, bioactive bands can be recovered from autobiograms followed by mass spectrometry analysis, facilitating the elucidation of the substances responsible for activity (14).

The objective of the present work was to demonstrate the power of effectively integrating chemical and biological approaches to better characterize food mixtures. For that, HPTLC coupled to bioassays was combined with LC-HRMS to identify substances responsible for the total estrogenic activity of food materials as measured by liquid format bioassays. Receptor-mediated estrogenic activity of soy protein isolates was selected as a relevant case study. Soy is known to contain a number of estrogenic active isoflavones (15–17), such as genistein, daidzein, glycitein, biochanin A, and formononetin. Extracts of soy isolates were tested in both the standard Chemical Activated LUciferase gene eXpression (CALUX) assay and in the HPTLC coupled to yeast-based assay (p-YES). Extracts and bioactive bands were also analyzed using a LC-HRMS method. Data were then integrated to get insight on the substances driving the estrogenic activity of soy protein isolates.

## Materials and methods

### Chemicals and materials

The Yeast Estrogen Screen (YES) McDonnell strains, the culture media and bioassay reagents, 10× McSD, 10×McDO, LacZ-Lysis buffer, 2-mercaptoethanol, and lyticase solution were purchased from Xenometrix, Allschwil, Switzerland. The substrate 4-methylumbelliferyl-β-D-galactopyranoside (MUG, CAS N°6160-78-7), 2-aminoethyl diphenylborinate (NP, CAS N°524-95-8) and polyethylene glycol 4,000 (PEG, CAS N°25322-68-3) were supplied by Sigma-Aldrich, Darmstadt, Germany. All solvents, HPLC grade quality, methanol, ethanol, chloroform, ethyl acetate, acetonitrile and water were purchased from Merck, Darmstadt, Germany. HPTLC plates Silica gel 60 (20cm × 10cm) and Silica gel 60 F254 (20cm × 10cm) were delivered by Merck, Darmstadt, Germany. Before testing, the plates follow a prewashing treatment to remove impurities by development in a twin trough chamber (20cm × 10cm, CAMAG, Muttenz, Switzerand) up to 5 mm from the top of the plate with methanol. After that, the plate was heated at 110°C during 15 min on the Plate Heater III (CAMAG, Muttenz, Switzerand).

Estrogen Receptor alpha (ERα) CALUX cell line was supplied by BioDetection System BV (Amsterdam, Netherlands) under license contract. For the cell maintenance and growth medium, DF- (D-MEM/F12 medium without phenol red as pH-indicator), DF + (D-MEM/F12 medium with phenol red as pH-indicator), FCS (Fetal Calf Serum) charcoal from Australian origin, MEM (100×) non-essential amino acids, PBS (phosphate buffered saline) pH 7.2; Ca2 + and Mg2 + free, G418 disulphate (CAS N°108321-42-2), Trypsin stock solution and Penicillin-streptomycin solution were supplied by Sigma-Aldrich, Darmstadt, Germany. RealTime-GloTM MT Cell Viability Assay was delivered by Promega AG, Dübendorf, Switzerland.

Soy Protein Isolate Pro Fam 974 (Soy Isolate 1) was obtained from ADM International Sarl (Rolle, Switzerland) and Soy Protein Isolate Supro (Soy Isolate 2) was provided by Solae Europe S.A. (Geneva, Switzerland).

Isoflavones compounds (Table 1) and reference compound, 17β-estradiol (E2, CAS N°6160-78-7) were purchased from Sigma-Aldrich, Darmstadt, Germany. QuEChERS salts (5,982–7,650) were provided by Agilent Technologies Inc., Basel, Switzerland.

**TABLE 1:** Isoflavones tested in the study.

Isoflavones	CAS no.	MW (g/mol)	Chemical structure
Genistein	446-72-0	270.24	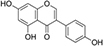
Genistin	529-59-9	432.38	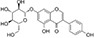
Daidzein	486-66-8	254.24	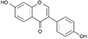
Daidzin	552-66-9	416.38	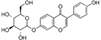
Glycitein	40957-83-3	284.26	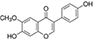
Glycitin	40246-10-4	446.4	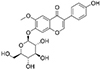
Biochanin A	491-80-5	284.26	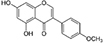
Naringenin	67604-48-2	272.25	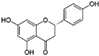
Formononetin	485-72-3	268.26	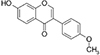

### Standard solutions

Stock solutions assays were prepared at 200 mM in DMSO. For HPTLC, standard solutions were obtained by dilution with ethanol. Positive control (E2) was prepared at a concentration of 0.3 pg/μL. Other standard solutions, genistein, genistin, daidzein, daidzin, glycitein, glycitin, and biochanin A, were prepared at a concentration of 10 ng/μL for HPTLC and 200 mM for CALUX. These solutions were stored at −20°C until use.

### Extraction and sample preparation

A modified QuEChERS extraction protocol without the cleaning step was applied to the two selected samples (18). In a 50-mL polypropylene tube, 1.0 ± 0.05 g of each selected soy isolate powder was added with a ceramic homogenizer and 10 mL of H_2_O. The extracts were shaken by hand to solubilize powder. Then, 10 mL of acetonitrile was added to each tube. The samples were shaken using the Geno-Grinder during 3 min at 1,500 rpm. QuEChERS salts, containing 4.0 g MgSO_4_, 1.0 g NaCl, 1.0 g Na_2_Ci, and 0.5 g Na_3_Ci, were added. After shaken using the Geno-Grinder during 3 min at 1,500 rpm samples were centrifuged at 4,000 × *g* during 10 min. In a 15-mL PTFE tube, 1.0 mL of the resulting supernatant were transferred and diluted 200 times in water before injection on a UPLC-MS/MS instrument. Another aliquot of 6.0 mL of supernatant was transferred in a 15-mL PTFE tube and evaporated to dryness under N_2_ at 40°C. The pellet was suspended in 1.6 mL of ethanol or 1.6 mL of H_2_O for HPTLC and CALUX bioassays, respectively. The extracts were stored at –20°C until use.

### Liquid chromatography tandem mass spectrometry

LC-MS/MS experiments were carried out using an Agilent 1,290 Infinity I LC platform (Agilent, Geneva, Switzerland) coupled to a QTrap 6500 + MS (SCIEX, Foster City, CA, USA). The chromatographic separation was performed on a BEH C18 column (100 × 2.1 (i.d.) mm; 1.7 μm) (Waters, Milford, MA, USA) maintained at 40°C with a flow rate of 400 μL/min. Mobile phase was composed of (A) 0.5 mM ammonium formate and 0.1% formic acid in water and (B) 0.5 mM ammonium formate and 0.1% formic acid in MeOH. The injection volume was set at 5 μL. The gradient started at 30% B and increased to 85% B over 5.5 min and then held at 100% B for 2 min before returning to initial conditions in 0.2 min and equilibrating for 2.2 min. The total run time was 10 min.

The mass analyzer was equipped with a Turbo V Ion Source and an ESI probe, operating in positive mode. MS parameters were obtained by infusing each individual compound (0.1 to 1 μg/mL) at a flow rate of 10 μL/min in line with mobile phases flow rate at 400 μL/min set at 50% organic mobile phase composition. The block source temperature (TEM) was maintained at 450°C and the gas set values were as follows: curtain gas 30 psi; nebulizer gas (GS1) 40 psi; turbo gas (GS2) 40 psi. Spray voltage was set at 4.5 kV. Optimum parameters for the multiple reaction monitoring (MRM) are summarized in Table 2.

**TABLE 2:** Optimal parameters for multiple reaction monitoring.

Analyte	Precursor ion	Ionization state	Production	DP^1^	EP^2^	CE^3^	CXP^4^	Dwell time (ms)
Genistein	271.1	(M + H) +	215.1 243.0	30 30	10 10	35 34	12 12	5
Genistin	433.0	(M + H) +	271.1 141.2	35 35	10 10	25 15	12 12	5
Daidzein	255.1	(M + H) +	199.1 137.2	25 25	10 10	34 35	12 12	5
Daidzin	417.2	(M + H) +	255.0 199.1	30 30	10 10	24 58	12 12	5
Glycitein	285.2	(M + H) +	270.0 242.0	40 40	10 10	35 42	12 12	5
Glycitin	447.2	(M + H) +	285.1 270.2	40 40	10 10	24 58	12 12	5
Biochanin A	285.2	(M + H) +	270.2 223.0	40 40	10 10	34 8	12 12	5

DP^1^: Declustering potential, EP^2^: Entrance potential, CE^3^: Collision energy, CXP ^4^: Collision cell exit potential.

Each sample was analyzed in duplicate. Each batch was carefully verified using quality control samples. Quantification was performed by means of an external calibration curve. Limit of quantification was 100 μg/kg for each of the seven isoflavones and recovery range from 68 to 113%.

### High Performance Thin-Layer Chromatography

Standard solutions and samples were applied (Automatic TLC Sampler 4, ATS4, CAMAG, Muttenz, Switzerland) as bands onto prewashed HPTLC Silica gel 60 or F254 plates with the following settings: band length 8 mm, dosage speed 80 nL/s, application volume between 1 and 15 μL, syringe installed 25 μL. The development was performed (Automatic Developing Chamber 2, ADC2, CAMAG) with a mixture of toluene/ethyl acetate/methanol/water/chloroform, 20:15:14.5:3.5:10 (V/V/V/V/V) up to 80 mm. After development, the plate was dried under cold air stream for 5 min.

### High Performance Thin-Layer Chromatography extracts chemical derivatization

A thin layer chromatography was performed to reveal the chemical profiles of the soy isolates extracts. The Silica gel 60 F254 plate was first documented under UV 254 nm light without chemical treatment with the TLC Visualizer 2 (CAMAG, Muttenz, Switzerland). Then, the plate was sprayed with 2 mL of a NP solution (1.0 g of 2-aminoethyl diphenylborinate in 100 mL methanol) using the Derivatizer (CAMAG) with green nozzle at level 4 and followed by a visualization at UV 366 nm. The second revelation was done with 2 mL of a PEG solution (5.0 g of PEG 400 in 100 mL ethanol) with blue nozzle at level 3. The bioautogram was documented under UV light 366 nm.

### High Performance Thin-Layer Chromatography coupled to YES assay (p-YES)

The applied protocol for the p-YES was based on previous publications (8–10) following some modifications based on the YES assay protocol supplied by Xenometrix. For the overnight (ON) culture of YES McDonnell strain, 19 mL of McDonnell growth medium (40 mL of sterile water, 5 mL of 10×McSD, and 5 mL of 10×McDO) were inoculated with 1 mL of yeast cells in a 500 mL glass sterile Erlenmeyer for 16 h at 31°C and 180 rpm. The ON culture was centrifuged (2,500 × *g*, 5 min) and resuspended in McDonnell growth medium to have a yeast suspension with 5.0 × 10^7^ cells/mL. The YES suspension (2 mL) was sprayed onto the plate with the Derivatizer using the red nozzle at level 6. Plate was incubated in a plastic box with nearly 100% relative humidity during 3 h at 31°C. The plate was dried 4 min under cold air stream after incubation. For the detection of β-galactosidase activity, a solution of MUG (5,850 μL of LacZ-Lysis buffer, 78 μL of 2-mercaptoethanol, 39 μL of lyticase and 40 μL of MUG 50 mg/mL in DMSO) was sprayed with the Chromajet DS20 (Biostep, Jahnsdorf, Germany). The following settings were selected: plate width 200 mm, plate length 100 mm, reagent quantity 4.01 mL, spray cycles 3. Plate was incubated in the plastic box with nearly 100% relative humidity during 15 min at 37°C. After that, the bioautogram was documented at UV 254 nm using TLC Visualizer. The MUG fluorescence was measured at 320/>400 nm with Deuterium lamp using TLC Scanner 4 (CAMAG).

### Chemical Activated LUciferase gene eXpression (CALUX)

The CALUX assay applied is based on the human osteoblast (U2-OS) cells genetically engineered (19) to express specific functional human ERα receptor under the control of specific luciferase responsive elements. An increase or decrease of signaling, results in corresponding changes in the expression of luciferase activity in the CALUX cells and is measured with a luminometer. Briefly, cells were seeded in a 96-well plate (VWR, Dietikon, Switzerland) and incubated for 24 h at 37°C, 5% CO_2_ and 100% humidity. Additionally, to the CALUX assay, cell viability was also assessed to detect cytotoxic effects. It was set-up and integrated in the dose-response analysis with the CALUX assay (20) as well as to the extracts using the RealTime GLO test from Promega.

### P-YES coupled to liquid chromatography high-resolution mass spectrometry (LC-HRMS)

Chemical analyses to identify compounds responsible for the estrogenic activity in the soy isolates samples were performed. For that, 15 bands of 5 μL of samples were applied on the plate followed by a chromatographic separation with the same protocol as described before. HPTLC bioactive bands at retention factor (Rf) 0.4 and 0.6 were manually recovered from 3 independent plates for the two soy extracts samples. The recovered bands were dissolved in ethanol, sonicated for 5 min and centrifuged for 5 min at 2,500 × *g* in duplicate. Supernatant was evaporated under N_2_ until dryness. A volume of 200 μL of water/methanol (90:10, v/v) was added to each dry extract, manually vortexed and ultrasonicated for 5 min. The solution was transferred into an Amicon Ultra-0.5 Centrifugal Filter Unit 3K (Merck, Darmstadt, Germany) and ultracentrifuged at 14,000 rpm at 4°C for 40 min. The eluate was collected and transferred in a vial before injection.

The liquid chromatography high-resolution mass spectrometry (LC-HRMS) method was performed as described by Bessaire et al. (21). The analyses were conducted on a Vanquish Horizon UHPLC system coupled to a Q-Exactive HF-X HRMS instrument (Thermo Fisher Scientific) controlled by TraceFinder 4.1 software (Thermo Fisher Scientific). The chromatographic separation was performed on a BEH C18 column (150 × 2.1 mm (i.d.), 1.7 μm) (Waters, Milford, MA, USA) heated at 50°C with a flow rate of 400 μL/min. The injection volume was 10 μL. Two chromatographic runs were considered either in alkaline or acidic conditions. Alkaline mobile phases consisted of water (mobile phase A) and methanol (mobile phase B) containing both 10 mM ammonium bicarbonate. The pH of mobile phase A was adjusted to 9.0 using ammonium hydroxide. A 25-min gradient was set as follows: 0–0.5 min (2% B); 0.5–14.5 min (40% B); 14.5–19.5 min (100% B); 19.5–22.0 min (100% B); 22.0–22.1 min (2% B); 22.1–25 min (2% B). Acidic mobile phases were water (mobile phase A) and methanol (mobile phase B) both containing 0.5 mM ammonium formate and 0.1% formic acid. A 22-min gradient was set as follows: 0–1.0 min (2% B); 1.0–18.0 min (100% B); 18.0–19.5 min (100% B); 19.5–19.6 min (2% B); 19.6–22.0 min (2% B). The Q-Exactive HF-X instrument was equipped with a heated-electrospray ionization (HESI-II) probe operating in positive or negative ionization modes (two separate injections). HESI-II and MS parameters were as follows: sheath gas and auxiliary gas flow rate 50 and 13 arbitrary units, respectively; sweep gas flow rate 3 arbitrary units, spray voltage 3.5 kV for the positive mode and 2.5 kV for the negative mode; capillary temperature 263°C; auxiliary gas heater temperature 425°C. Automatic gain control (AGC) target value was set at 3 × 10^6^ ions and maximum injection time (IT) at 50 ms with resolving power of 120,000 FWHM (at m/z = 200) in full scan MS over the range 90–1,050 Da. Data dependent fragmentation (dd-MS2) was set with a resolving power of 15,000 FWHM (at *m/z* = 200), isolation window of 1.0 Da, AGC of 1 × 10^5^ ions, maximum IT of 50 ms, dynamic exclusion duration of 3 s and intensity threshold of 2 × 10^4^ cps. An inclusion list of selected parent ions was used with optimized normalized collision energy (NCE).

### Data analysis

The data obtained using the ERα CALUX assay were reported as Relative Induction (RI) by normalization of the maximum Relative Luminescence Units (RLU’s) signal of the reference compound (E2) to 100%. The tested compounds were then expressed as a percentage of the maximum reference compound response. All dose-responses and dilutions were performed in technical triplicates.

The HPTLC biodensitograms were evaluated using the peak height by integrating each peak with the software VisionCats 3.0 (CAMAG). Data were analyzed using the average of triplicates performed for each pure compound and soy extract considering the dose response effect. The solvent control and reference compound (E2) were applied in each plate to confirm the validity of the test. Twenty peaks in the solvent control track were used as the mean blank sample. The results were expressed as a percentage of the maximum activity by normalizing the maximum peak height signal obtained from the reference compound (E2) to 100%. Data graphs were produced using GraphPad Prism 9.0.0 (GraphPad Software LLC, San Diego, CA, USA).

For the analytical data for recovered bands, differential analyses (Rf 0.4 and Rf 0.6 vs. silica blank) were performed with Compound Discover 3.0 software. Results were filtered using *p*-value (<0.01), area ratio between groups (>4) and identification from Arita lab Flavonoids list (containing 6,549 flavonoids). Signals were identified using in-house and web-based spectral libraries, accurate mass, and retention time.

### Quantification of formononetin and naringenin

QuEChERS extraction was performed as described above with the modification that the supernatant was transferred into a PTFE tube and evaporated to dryness before reconstitution in Water/Methanol (9:1, v/v). The solutions were then transferred into a snap-lock tube and centrifuged at 17,000 × *g* for 20 min at 4°C using a centrifuge Heraeus Frisco 17 (Thermo Scientific) to facilitate precipitation of remaining impurities. The upper phase was transferred into LC vials and injected using the method described before. Quantification was performed using the standard addition as described in Bessaire et al. (21). The samples were analyzed in duplicate: as such (i.e., unspiked test portion) and fortified 500 μg/kg and 5,000 μg/kg for formononetin and naringenin, respectively. The level of both analytes was estimated based on a one-point standard addition quantification approach.

### Interpolation of estrogenic activities

Interpolation calculation was applied to estimate the contribution of isoflavone in estrogenic activity of the soy protein isolates. Estrogenic activity interpolation was calculated from specific dose-response available for each substance and their quantity using non-linear sigmoidal regression and the command interpolate unknowns from standard curve at confidence interval of 95% (GraphPad Software LLC, San Diego, CA, USA).

## Results

### Estrogenic activity of soy protein isolates

**FIGURE 1:**
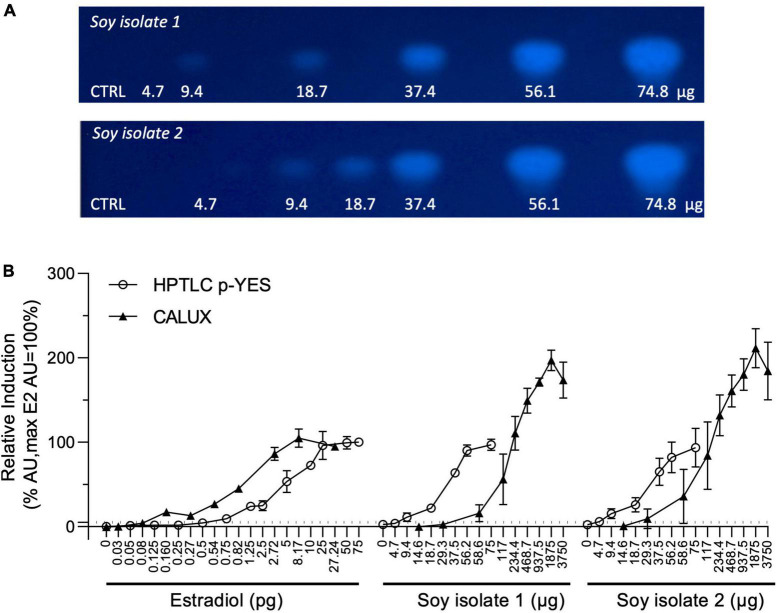
CALUX and p-YES estrogen receptor activation of soy isolates extracts. **(A)** Bioautograms of the two soy isolates under UV-light 366 nm after p-YES assay (region of interest shown). **(B)** Dose-response curves obtained with CALUX and HPTLC p-YES. CALUX results are expressed in μg or pg per well, HPTLC results are expression in μg or pg per band. The ERα threshold above which a response is considered positive is indicated with a black dashed line.

Two soy isolates were extracted using the QuEChERS method without the cleaning step. Indeed, the cleaning step is impacting both, the sample recovery and a loss of bioactive substances [e.g., estrogenic substances (data not shown)]. The soy extracts were tested for estrogenic activity using CALUX assay and HPTLC p-YES. For both bioassays, dose-response curves from extracted samples were obtained in triplicate. As positive control, estradiol was used in each experiment, from 0.03 to 27.24 pg/well and at 5 pg/band for CALUX and p-YES assays, respectively. Transcription activation of estrogen activity was recorded in the two soy isolates with both bioassays. A clear bioactive zone with a dose-response effect was observed with p-YES (Figure 1A). Figure 1B depicts the dose-responses as percentage of activity using the estradiol as reference. It also presents the estrogenic activity obtained with the soy protein isolate extracts in the standard CALUX assay.

### Chemical analytical detection of isoflavones in soy protein isolates

Soy is well-documented to contain estrogenic isoflavones (15, 17). These substances are anticipated to be present in the tested isolates and are considered to drive the measured estrogenic activity in these materials. To confirm this hypothesis, the two soy isolates were analyzed by LC-MS with the aim to identify and quantify the main isoflavones. Seven isoflavones were identified: genistein, genistin, daidzein, daidzin, glycitein, glycitin, and biochanin A. Quantities found in both extracts are summarized in Table 3.

**TABLE 3:** Isoflavone levels in soy isolates as measured by LC-MS/MS.

	Soy isolate 1	Soy isolate 2
**Isoflavones**	**μg/kg**	
Genistein	8 595	9 779
Genistin	15 533	24 310
Daidzein	3 968	7 536
Daidzin	3 956	8 440
Glycitein	503	1 307
Glycitin	620	2 303
Biochanin A	<100 (10)	<100 (20.3)

### Compound identification and quantification in bioactive bands

**FIGURE 2:**
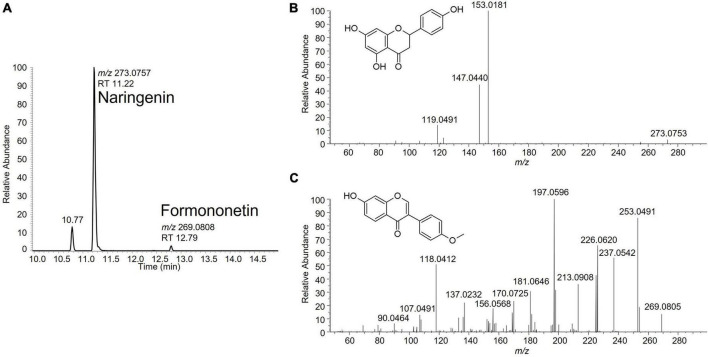
**(A)** Extracted ion chromatogram for naringenin (C_15_H_12_O_5_; *m/z* 273.0757) and formononetin (C_16_H_12_O_4_; *m/z* 269.0808) in a soy protein isolate sample. **(B)** Fragmentation spectrum of naringenin at NCE 50. **(C)** Fragmentation spectrum of formononetin at NCE 70.

A suspect analytical screening strategy was applied on the bioactive band recovered from the HPTLC plate to assign estrogenic activity to individual compound(s) (8–10). The samples were analyzed by liquid chromatography coupled to high-resolution mass spectrometry (LC-HRMS). Several signals potentially linked to estrogenic flavonoids were highlighted. Some signals were attributed to previously identified isoflavones such as genistein, glycitein and daidzein, known for their estrogenic activity (11). Two additional signals were tentatively identified as naringenin and formononetin based on comparison of their fragmentation data with in-house and publicly available spectral databases (Figure 2). Their identity was eventually confirmed by injection of reference standards. Following their identification, the levels of naringenin and formononetin in the soy isolates 1 and 2 were estimated using a one-point standard addition quantification approach. Naringenin levels were 5,250 and 6,700 μg/kg while formononetin was detected at 53.6 and 64.2 μg/kg in soy isolates 1 and 2, respectively.

### HPTLC-based detection of isoflavones in soy protein isolates

**FIGURE 3:**
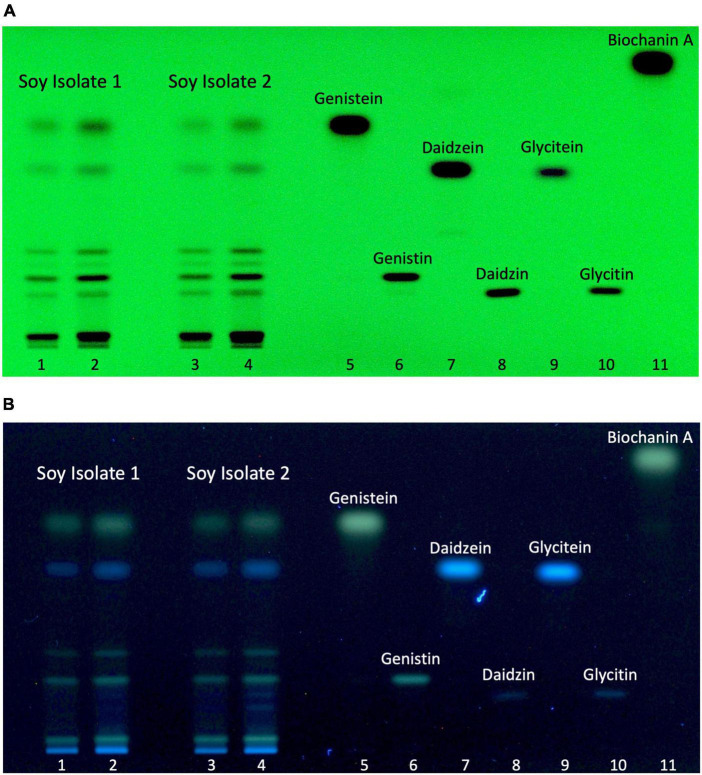
Soy isolate isoflavone profiles. Soy isolate 1 (4 and 6 μL, line 1 and 2, respectively), soy isolate 2 (4 and 6 μL, line 3, and 4, respectively), genistein (1.3 μg, line 5), genistin (1 μg, line 6), daidzein (1.3 μg, line 7), daidzin (1 μg, line 8), glycitein (0.4 μg, line 9), glycitin (0.6 μg, 10), and biochanin A (1 μg, line 11). **(A)** Autogram under UV-light 254 nm. **(B)** Autogram after chemical revelation with NP/PEG and documentation under UV-light 366 nm.

The possibility to identify and discriminate isoflavones in HPTLC-plates was ascertained by applying generic standard detection methods including UV-light (254 nm) exposure without chemical reagent (Figure 3A) and UV-light exposure (366 nm) after derivatization with NP/PEG (Figure 3B). Relevant isoflavones were revealed in both extracts. Discrete black bands were visible under UV-light at 254 nm. The use of chemical standards allowed for the assignment of 6 bands to specific isoflavones as measured by chemical analysis. The isoflavones most prevalent in the two samples were genistein and its glycoside genistin, which were correlated with levels found by LC-HRMS. When applying UV-light (366 nm) after NP/PEG derivatization, the three isoflavones (genistein, genistin, and biochanin A) with a hydroxyl group on the C5 appeared as green bands, while the others appeared as blue color. Essentially, both revelation methods provided similar data, indicating that the HPTLC conditions can separate the different isoflavones present in the isolates.

### Estrogenic activity of isoflavones

**FIGURE 4:**
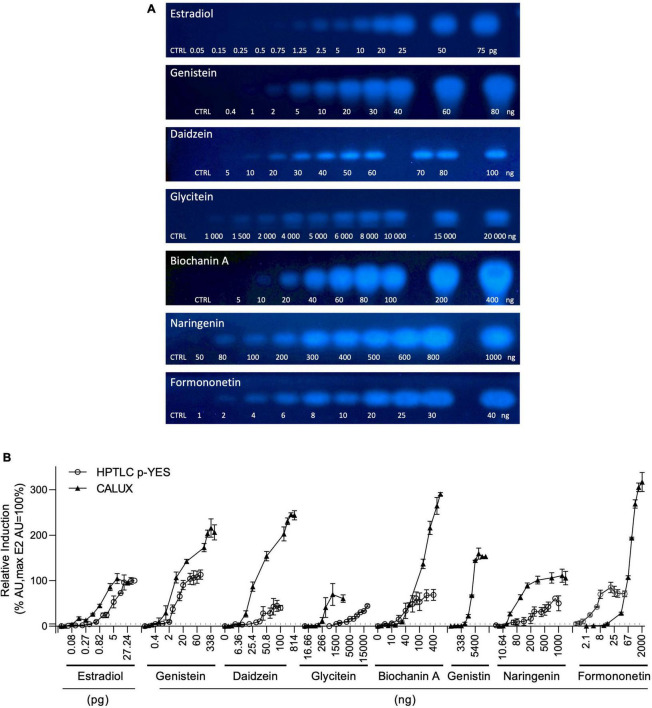
p-YES and CALUX estrogen receptor activation of isoflavones identified in soy protein isolates. **(A)** Bioautograms of isoflavones under UV-light 366 nm after p-YES assay (region of interest shown). **(B)** Dose response curves obtained with CALUX (black triangle) and HPTLC p-YES (black circle). For liquid format, data are expressed in pg or ng/well. For p-YES, data are expressed in pg or ng/band. The ERα threshold above which a response is considered positive is indicated with a black dashed line.

The estrogen receptor (ERα) mediated activity of the seven isoflavones identified was evaluated using the standard CALUX and the HPTLC-pYES assays. The dose-response curves for each isoflavone were obtained in three independent experiments using biological triplicates. Estradiol was selected as a reference compound. For the CALUX assay, the dose tested for each isoflavone was adjusted based on preliminary experiments (0.16–675.6 ng/well for genistein; 84.4–43,200 ng/well for genistin; 1.59–814 ng/well for daidzein; 162.65–83 276 ng/well for daidzin; 16.66–2,140 ng/well for glycitein; 43.59–22,320 ng/well for glycitin; 3.56–910 ng/well for biochanin A; 5.32–2,000 ng/well for naringenin and 2.1–2,000 ng/well for formononetin). For the p-YES, dose-responses were also generated (0.4–80 ng/band for genistein; 10–2,000 ng/band for genistin; 5–100 ng/band for daidzein; 1,000–20,000 ng/band for glycitein; 250–2,000 ng/band for daidzin and glycitin; 5–200 ng/band for biochanin A; 50–1,000 ng/band for naringenin and 1–40 ng/band for formononetin). For both tests, the obtained signals were normalized to the upper signal of estradiol to calculate the percentage of activity (Figure 4B). With both assays, no estrogenic activity was observed for daidzin and glycitin (data not shown). A clear dose-response effect was obtained for estradiol and isoflavones with both p-YES (Figures 4A, B) and CALUX (Figure 4B). In the two assays, a wide estrogenic activity potency range was observed according to the different isoflavones (Table 4).

**TABLE 4:** EC_50_ of the CALUX assay and HPTLC p-YES.

Chemical	CALUX	p-YES
Estradiol (pg)	1.04	5.5
Genistein (ng)	6.2	8.5
Genistin (ng)	2 975	nd
Daidzein (ng)	36.9	49.2
Daidzin (ng)	nd	nd
Glycitein (ng)	447.8	9 282
Glycitin (ng)	nd	nd
Biochanin A (ng)	125.7	41.5
Naringenin (ng)	71.4	432.9
Formononetin (ng)	102	5.6

Results are expressed in pg or ng/well and in pg or ng/band for CALUX and HPTLC, respectively. nd: not detectable.

### Identification of substances responsible for estrogenic activity of soy protein isolates

The bioassay data combined with chemical analyses allowed for the identification of 4 isoflavones (genistein, daidzein, naringenin and formononetin) exhibiting substantial estrogenic activity. To get insight into which of these molecules could contribute the most to the overall estrogenic activity of the soy protein isolates, an interpolation calculation was applied. In a first step, the levels of the isoflavones quantified by LC-MS/MS in the isolates (μg/kg, Table 3) were used to estimate the concentrations available in the bioassays from exposure to the maximum possible amounts of samples as expressed in isolate mass (3.75 mg). In the second step, the isoflavone concentrations were converted into estrogenic activity equivalents (relative induction, RI) using the specific dose-response available for each substance. The interpolated RIs for the amounts of substances present in 3.75 mg of isolates are shown in Table 5. Interpolated activities indicate that genistein and daidzein, and to a lesser extend naringenin, are the drivers of the soy protein isolate estrogenic activity measured in the standard CALUX assay. Therefore, at the conditions applied, the observed p-YES bioactive zone in the autobiogram is explained by the presence of genistein, daidzein, and naringenin at the same Rf’s zone (Rf’s between 0.6 and 0.76) as tested using the HPTLC derivatization using NP/PEG as described before (data not-shown).

**TABLE 5:** Interpolated induction response of isoflavones present in soy protein isolates.

			CALUX		HPTLC	
	**Soy isolate 1**	**Soy isolate 2**	**Soy isolate 1**	**Soy isolate 2**	**Soy isolate 1**	**Soy isolate 2**
**Isoflavones**	**ng/3.75 mg**		**Interpolated Relative Induction**			
Genistein	32.23	36.67	(150–173)	(154–177)	(98–108)	(100–110)
Genistin	58.24	91.16	<min	(0–6)	na	na
Daidzein	14.88	28.26	(34–47)	(86–100)	(0–12)	(0–14)
Daidzin	14.84	31.65	na	na	na	na
Glycitein	1.89	4.90	<min	<min	<min	<min
Glycitin	2.33	8.64	na	na	na	na
Biochanin A	0.04	0.08	<min	<min	<min	<min
Naringenin	19.69	25.13	(1–11)	(5–16)	<min	<min
Formononetin	0.20	0.24	<min	<min	<min	<min

Interpolated Relative Induction (RI) is given with a range of minimal to maximal expected responses. na: not applicable (no activity detected); <min: below limit of quantification.

## Discussion and conclusion

There is an increasing concern regarding the presence of substances with endocrine disruption properties in food. Two different strategies are being promoted for their identification and detection. The first focuses on analytical chemistry. It is very powerful to detect and quantify known substances and is considered the approach of choice in view of risk assessment and management. But analytical chemistry is not designed to assign biological properties to molecules and is therefore limited as a tool to identify unknown endocrine active chemicals in complex mixtures such as food materials. A second, emerging strategy uses bioassays as analytical detectors (1, 2, 4). Testing a complex mixture in a bioassay is thought to provide potential benefits such as revealing the presence of not only known but also unknown bioactive substances. Additionally, it may contribute to the identification of possible interaction effects between different substances. However, bioassays alone do not allow for the determination of the components responsible for the activity and, therefore, are not appropriate to characterize the significance of measured activity for endocrine disruption and health risks. The integration of analytical chemistry and bioassay data is expected to bring synergisms in the investigation and understanding of endocrine properties of complex mixtures such as food materials.

The objective of the present work was to investigate how HPTLC coupled to the pYES-bioassay and LC-HRMS could contribute to the elucidation of chemicals responsible for the ERα-mediated estrogenic activity of a food material. For this, soy protein isolates were chosen because of their anticipated estrogenic activity from naturally occurring isoflavones. As expected, extracts of soy protein isolates produced significant estrogenic activity in the ERα-CALUX assay. The same extracts were tested in the HPTLC coupled to estrogenic assay (p-YES). A strong fluorescent bioactive zone was visible on the autobiograms suggesting either (1) the presence of a strong estrogenic substance in the extracts, or (2) several estrogenic compounds present at similar Rf values and/or (3) some isoflavones do not trigger estrogenic activity or are present at undetectable levels.

Chemical analysis of the isolates demonstrated the presence of several isoflavones/flavonoids, some of them known to be estrogenic. Amounts measured were highly variable according to individual substances, with levels ranging over 3 orders of magnitude. The use of different derivatization methods confirmed that the HPTLC conditions applied resulted in a separation of these molecules. Testing each individual substance in CALUX and p-YES bioassays identified 6 of them exhibiting measurable estrogenic activity in both tests. Variable potencies were observed according to individual substances, with some differences of ranking depending on the bioassay applied. Taken together, these data indicate that the intensity of bands visible on the p-YES plate depends on a combination of both substance-specific levels and estrogenic potencies. These two factors most likely explain the observation that a single bioactive zone is visible in the p-YES analysis of soy protein isolates. Genistein is both potent and present at high levels in the isolates, leading to the production of a very strong signal and, therefore, it was easily visible. The other isoflavones may not be visible because of either too low levels (e.g., biochanin A) or weak potencies (e.g., glycitin), or both (e.g., glycitein). Importantly, a strong signal may drive the visualization conditions of the test and consequently mask the presence of other, fainter bands. This type of interference will have to be addressed in further research.

Suspect screening analysis of the bioactive band highlighted several signals and provided significant information toward the characterization of the estrogenic activity of the soy protein isolates. Some signals were attributed to previously identified isoflavones such as genistein, glycitein and daidzein, known (and confirmed by the present work) for their estrogenic activity (11). Furthermore, two additional active substances (naringenin and formononetin), not considered in the initial list of chemicals to be analyzed, were detected, illustrating the potential of p-YES coupled to LC-HRMS to identify unforeseen, but potentially relevant active substances.

The integration of all data from the three methods applied allowed for the elucidation of the nature of the chemicals responsible for the estrogenic activity measured in the extracts of the soy protein isolates. Genistein and daidzein are contributing the most while naringenin could play a small role. As hypothesized above, other isoflavones are not expected to have any impact because of either low concentrations or weak estrogenic potencies, or both. This case study demonstrates the potential of an integrated approach using CALUX and HPTLC-pYES bioassays together with analytical chemistry to decipher estrogenic activity of complex matrices such as food materials.

To analyze and characterize endocrine activity of complex mixtures, such as food materials or packaging migrates, is of increasing priority. To address this need, bioassays are thought to play an important role. But to be able to interpret bioassay data from a safety perspective and, if needed, to allow for the development of management options requires understanding the origin of the activity. This is not straightforward. Based on the present work, the following strategy can be proposed to fully characterize the endocrine activity of a complex mixture:

1. Qualified sample preparation procedure (avoid cleaning step as it may remove endocrine and probably other type of bioactive substances).
2. Assess overall activity in a bioassay in liquid format. This requires using a validated bioassay.

1. In parallel, test the same sample in HPTLC-bioassay.
2. Run suspect analysis of the extracts and bioactive zones. Identify possible active substance candidates. This can be done either by literature searches or through the computational modeling of ligand-protein interactions (22).
3. For the identified candidates, generate dose-response curves in bioassays.
4. Quantify the most likely candidates in the samples.
5. Using the activity dose-response curves for the likely candidates, convert the analytical data into activities and determine the key contributors to the overall activity of the sample under investigation.

The strategy, as highlighted above, is currently applicable to a limited number of endocrine activities for which HPTLC hyphenated bioassay methods are available. Interestingly, such an approach can be developed for other fields where the question of bridging analytical data to bioactivity is critical.

## Data availability statement

The original contributions presented in this study are included in this article, further inquiries can be directed to the corresponding author.

## Author contributions

MM-K contributed to the methodology of the study. ED, BG, HL, PS, NC, ME, and TB performed the investigations. ED, BG, and HL wrote the first draft of the manuscript. ED, HL, BG, FB, and NC performed the visualization. MM-K and FB supervised the project. All authors contributed to the manuscript revision, read, and approved the submitted version.
